# Comparing the effectiveness and safety of videolaryngoscopy and direct laryngoscopy for endotracheal intubation in the paediatric emergency department: a systematic review and meta-analysis

**DOI:** 10.3389/fmed.2024.1373460

**Published:** 2024-09-19

**Authors:** Emma Warinton, Zubair Ahmed

**Affiliations:** ^1^Institute of Inflammation and Ageing, College of Medical and Dental Sciences, University of Birmingham, Birmingham, United Kingdom; ^2^Centre for Trauma Sciences Research, University of Birmingham, Birmingham, United Kingdom; ^3^Surgical Reconstruction and Microbiology Research Centre, National Institute for Health Research Queen Elizabeth Hospital, Birmingham, United Kingdom

**Keywords:** laryngoscopy, intubation, airway management, child, pediatrics, emergency medicine, systematic review

## Abstract

**Introduction:**

Endotracheal intubation is an uncommon procedure for children in the emergency department but can be technically difficult and cause significant adverse effects. Videolaryngoscopy (VL) offers improved first-pass success rates over direct laryngoscopy (DL) for both adults and children undergoing elective surgery. This systematic review was designed to evaluate current evidence regarding how the effectiveness and safety of VL compares to DL for intubation of children in emergency departments.

**Methods:**

Four databases (MEDLINE, Embase, CENTRAL and Web of Science) were searched on 11^th^ May 2023 for studies comparing first-pass success of VL and DL for children undergoing intubation in the emergency department. Studies including adult patients or where intubation occurred outside of the emergency department were excluded. Quality assessment of included studies was carried out using the Risk Of Bias In Non-randomised Studies of Interventions (ROBINS-I) tool. Meta-analysis was undertaken for first-pass success and adverse event rate.

**Results:**

Ten studies met the inclusion criteria representing 5,586 intubations. All included studies were observational. Significantly greater first-pass success rate was demonstrated with VL compared to DL (OR 1.64, 95% CI [1.21–2.21], *p* = 0.001). There was no significant difference in risk of adverse events between VL and DL (OR 0.79, 95% CI [0.52–1.20], *p* = 0.27). The overall risk of bias was moderate to serious for all included studies.

**Conclusion:**

VL can offer improved first-pass success rates over DL for children intubated in the emergency department. However, the quality of current evidence is low and further randomised studies are required to clarify which patient groups may benefit most from use of VL.

**Systematic review registration:**

https://www.crd.york.ac.uk/prospero/display_record.php?RecordID=415039, Identifier CRD42023415039.

## 1 Introduction

Endotracheal intubation (ETI) is an uncommon but critical procedure for children in the emergency department. ETI may be indicated to secure the airway in both medical and trauma patients, such as in cases of respiratory failure, seizure, head injury, or cardiac arrest (1, 2). Potential adverse events following intubation include: oesophageal intubation, hypoxaemia, aspiration, tissue trauma, and failure to secure the airway (3). ETI can be life-saving but technically difficult in children due to how their anatomy and physiology differs from adults (4). A child’s relatively larger head, shorter neck, and larger tongue increase the likelihood of difficult laryngoscopy in this population. Greater oxygen consumption coupled with a reduced functional residual capacity can cause children to rapidly desaturate while apnoeic during laryngoscopy, despite pre-oxygenation. There are also fewer opportunities for clinicians to practise paediatric airway management given that critical illness is far less common than in the adult population and emergency ETI is only required in a small proportion of paediatric presentations (2–33 per 10,000 visits) (1, 2, 5). Multiple intubation attempts are associated with increased risk of adverse airway outcomes (2), therefore interventions and techniques which optimise first-pass success are crucial.

In recent years, there has been a dramatic increase in the availability and usage of videolaryngoscopy (VL) devices to aid ETI (6, 7). A camera attached to the tip of the blade allows for indirect visualisation of the glottis without requiring a direct line of sight. Videolaryngoscopes based on Macintosh blades (e.g., C-MAC [Karl Storz]) can be used for direct and indirect laryngoscopy interchangeably, whereas those with hyperangulated blades (e.g., GlideScope [Verathon Inc.]) offer indirect visualisation only (6). Greater angulation of the blade allows for visualisation of the glottis without the degree of neck extension required for traditional direct laryngoscopy (DL). VL is also beneficial for teaching and supervision of ETI and allows the procedure to be recorded for quality assurance, research, or education purposes (7). Studies have demonstrated a faster learning curve with VL devices, resulting in novice operators achieving higher success rates (6). This could be particularly beneficial for infrequent but critical procedures such as paediatric intubation (5). VL footage could even be streamed by clinicians remotely, facilitating supervision of ETI in rural or pre-hospital settings (8).

VL has been incorporated into airway management guidelines for many countries, particularly regarding management of patients with coronavirus disease 2019 (COVID-19) (9–11). These recommendations focus on minimising the number of attempts and time to intubation in difficult airway situations rather than the explicit use of VL as a first-line approach, given that operator experience and expertise are key factors in success with VL devices. The current evidence, however, yields mixed conclusions on the effectiveness of VL compared to DL, varying by setting, device, and patient and operator characteristics. For example, Hansel et al. concluded that VL offers superior first-pass success and glottic visualisation over DL in adults, and in particular, devices with hyperangulated blades were associated with lower rates of oesophageal intubation and greater intubation success in difficult airways (12). Among paediatric patients, randomised controlled trial (RCT) evidence found improved glottic view and significantly lower rate of failed first intubation attempt with VL (13). In both of these studies, the majority of procedures were undertaken in elective operating theatre settings (12, 13), with mixed results seen in adult studies set in emergency departments and intensive care units. Perkins et al. reported improved first-pass success with VL compared to DL in 13/23 (56.5%) studies outside the operating theatre, with the remainder showing no significant difference (10). These studies show how VL was associated with improved glottic views and reduced rates of oesophageal intubation in all studies where this outcome was reported. All of these studies highlight heterogeneity as a limitation on evidence quality, particularly in emergency settings where there is a paucity of RCT evidence and thus a greater reliance on observational data. Consensus is needed on how VL can best be utilised in these different settings, with clear guidelines for specific patient and operator groups.

To our knowledge, there are no systematic reviews comparing VL and DL for paediatric patients in an emergency department setting. Hence, this systematic review aimed to appraise the available evidence comparing the effectiveness of VL and DL for children undergoing ETI in emergency departments and pool study data as appropriate.

## 2 Methods

This systematic review was registered with PROSPERO (CRD42023415039; https://www.crd.york.ac.uk/prospero/display_record.php?RecordID=415039) and was reported in line with the Preferred Reporting Items for Systematic Reviews and Meta-Analysis (PRISMA) recommendations (14).

### 2.1 Eligibility criteria

Inclusion criteria for eligible studies were established prior to database searches based on a PICO (Population Intervention Comparator Outcome) framework. The population was defined as paediatric patients undergoing ETI in the emergency department; the intervention was VL with any device; the comparator was DL with any blade type; and the primary outcome of interest was first-pass success (FPS). Trials were only included where FPS data was reported for both VL and DL groups. Studies including patients >18 years of age, mannequin studies, and studies where intubation occurred outside the emergency department were excluded. RCTs and observational studies were included as a lack of RCTs was anticipated based on scoping searches. Case reports, case series, conference abstracts, and correspondence were excluded.

### 2.2 Information sources and search strategy

A systematic literature search of four databases was conducted on 11^th^ May 2023: MEDLINE, Embase, Cochrane Central Register of Controlled Trials (CENTRAL), and Web of Science. Search strategies were devised based on the PICO framework and included keywords and MeSH terms relating to “intubation,” “videolaryngoscopy,” “paediatrics,” and “emergency department” (see Appendix 1 for detailed strategies for each database). Searches were limited to English language only. Date restrictions were not applied.

### 2.3 Selection process

Two reviewers (EW and ZA) independently screened the titles and abstracts of identified studies. Discrepancies were resolved by discussion and mutual agreement. Full texts of relevant articles were retrieved and compared to the inclusion criteria. Rayyan software was used to collate database search results, remove duplicate articles, and screen abstracts (15). Potential duplicates were identified automatically and confirmed by a reviewer (EW).

### 2.4 Data collection

Data was extracted by reviewer EW and checked by reviewer ZA. Discrepancies were resolved by discussion and mutual agreement. Data were collected on country of study, setting of study, study design, dates of data collection, age of participants, total number of participants, type of videolaryngoscope used, and study outcomes. For participants undergoing VL or DL for intubation, the following data points were collected: number of participants in each group, number of FPS intubations, number of adverse events, and calculated effect sizes (e.g., odds ratios) for any outcomes. Where adjusted odds ratios for either outcome were reported, the factors adjusted for were documented.

### 2.5 Risk of bias assessment

The Cochrane Risk Of Bias In Non-randomised Studies of Interventions (ROBINS-I) tool was used to assess the methodological quality of the included studies (16). Studies were assessed for risk of bias in the following domains: confounding, selection of participants, classification of interventions, deviations from intended interventions, missing data, measurement of outcomes, and selection of the reported result. The risk of bias was judged to be low, moderate, serious, or critical in each domain and for the study overall. Overall quality was judged as low for studies with low risk of bias in all domains, moderate for studies with low or moderate risk of bias in all domains, serious for studies with serious risk of bias in at least one domain, and critical for studies with critical risk of bias in at least one domain. Quality assessment was undertaken independently by two reviewers (EW and ZA) and discrepancies were resolved by discussion and mutual agreement.

### 2.6 Effect measures & statistical analysis

FPS and adverse event data are presented as percentage rates and odds ratios with 95% confidence intervals. Individual study data was combined for unadjusted FPS rates as the primary outcome, and adverse event rates as a secondary outcome for studies where these data were available. A random-effects model was used to account for variance both within and between studies, and data were analysed using the DerSimonian and Laired inverse variance method (17). The meta-analysis was carried out using Review Manager 5 (RevMan 5, Version 5.4, The Cochrane Collaboration, Copenhagen). Meta-analysis results are presented in forest plots as odds ratios with 95% confidence intervals and pooled overall effect measures. Statistical heterogeneity was assessed using the I^2^ methodology, with values >50 and >75% taken to indicate moderate and significant heterogeneity between studies, respectively. All *p*-values were two-tailed and considered statistically significant if <0.05. Formal subgroup analyses for age group and operator experience were not carried out as per the protocol due to heterogeneity in study methodology limiting comparability of data in these groups.

## 3 Results

### 3.1 Study selection

**Figure 1:**
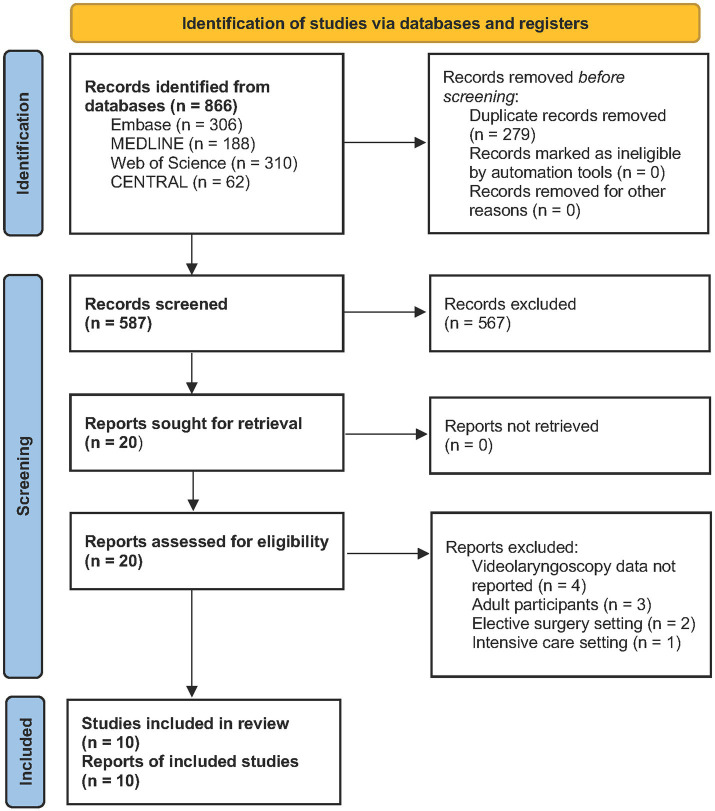
PRISMA flowchart of study selection.

Database searches yielded 866 results, of which 279 were identified as duplicates and removed (Figure 1). Five hundred and eighty seven titles and abstracts were screened, leaving 20 full text articles to be assessed for eligibility. Ten articles were excluded due to intensive care setting (*n* = 1), elective surgery setting (*n* = 2), adult participants included (*n* = 3), or VL data not reported (*n* = 4). Ten articles were assessed to be eligible and were included in the review (18–27).

### 3.2 Study characteristics

The eligible studies included a total of 5,886 participants, of whom 2,436 underwent VL and 3,034 DL for ETI in the emergency department (see Table 1). All studies were observational: two were retrospective reviews of medical records (18, 22), seven used prospectively-collected data (19, 21, 23–27), and one study compared prospective cases to retrospective controls (20).

**Table 1:** Characteristics of included studies.

Author	Data collection	Study design	Country	Setting	Total no. of participants	Population	VL device	Primary outcome	Secondary outcome(s)
Abid et al. (18)	2004–2018	Retrospective observational	US	Tertiary care paediatric ED	628	<18 yrs	C-MAC	Number of intubation attempts	Intubation-associated adverse events
Choi et al. (19)	2006–2010	Prospective observational	South Korea	Multicentre academic EDs	281	<10 yrs	Not specified	First-pass success rate	Adverse events
Couto et al. (20)	2016–2018	Prospective observational with retrospective controls	Brazil	Academic paediatric tertiary centre ED	191	1-18 yrs	McGrath Mac	First-pass success rate	Desaturation, intubation-associated events
Donoghue et al. (21)	2016–2020	Prospective observational	US	Multicentre tertiary children’s hospital EDs	494	<18 yrs	C-MAC	Tracheal intubation success	Time of laryngoscopy, hypoxaemia
Eisenberg et al. (22)	2004–2014	Retrospective observational	US	Tertiary care paediatric ED	439	<18 yrs	C-MAC	First-pass success rate	Successful intubation, complication rate
Ghedina et al. (23)	2010–2015	Prospective observational	Australia/ New Zealand	Multicentre EDs	270	<16 yrs	Not specified	First-pass success rate	Complication rate
Kaji et al. (24)	2016–2018	Prospective observational	International (NEAR)	Multicentre academic & community EDs	625	<16 yrs	Any VL device	First-pass success	Adverse events
Miller et al. (25)	2017–2021	Prospective observational	US/Canada	Multicentre academic paediatric EDs	1,412	<18 yrs	Not specified	First-pass success	Adverse airway outcomes
Pacheco et al. (26)	2007–2017	Prospective observational	US	Tertiary care paediatric ED	493	<18 yrs	C-MAC/ GlideScope	First-pass success without adverse events	Adverse events
Pallin et al. (27)	2002–2012	Prospective observational	International (NEAR)	Multicentre academic & community EDs	1,053	<16 yrs	Not specified	First-pass success	Adverse events

VL, videolaryngoscopy; ED, emergency department; NEAR, National Emergency Airway Registry.

The majority of trials were based in North America (18, 21, 22, 25, 26), with other studies from South Korea (19), Brazil (20), and Australasia (23). Six studies utilised multicentre data (19, 21, 23–25, 27), including two which took cases from the National Emergency Airway Registry (NEAR), an international network of hospitals with most centres in the US (24, 27). The dates of data collection of these two studies were mutually exclusive, therefore there should be no duplicated cases. All single-centre studies were based in tertiary care centres (18, 20, 22, 26). Length of the data collection period varied across studies, ranging from 2–14 years. Five studies reported use of particular VL devices: three studied C-MAC (18, 21, 22), one studied both C-MAC and GlideScope (26), and one studied McGrath MAC (20). FPS was the most common primary outcome, with the majority of studies reporting adverse events as a secondary outcome. One study however, reported the use of VL on only 5 participants as opposed to 276 patients that were intubated using DL and we draw caution to the interpretation of this study (18).

### 3.3 Results of individual studies

FPS was reported for VL and DL in all included studies (Table 2). FPS rate ranged from 20.0 to 94.1% for VL and 37.6 to 82.8% for DL. However, nine of the 10 studies reported FPS rates using VL of >68% with only one study reporting an FPS rate of 20%, most likely due to the extremely low number of participants in this group, with one successful attempt out of 5 participants (18). Three studies reported unadjusted odds ratios for FPS (22, 24, 25), with two studies (66.7%) finding significantly greater odds of FPS with VL than DL (OR 1.80, 95% CI 1.20–2.70; OR 2.05, 95% CI 1.56–2.70) (24, 25). 4/5 (80%) studies demonstrated significantly greater odds of FPS with VL than DL, when adjusting for various confounding factors (20, 24–26).

**Table 2:** First-pass success data.

Author	Total VL participants	Total DL participants	VL FPS participants	DL FPS participants	VL FPS rate	DL FPS rate	FPS OR (unadjusted)	FPS OR (adjusted)
Abid et al.	381	244	274	175	71.9%	71.7%	-	-
Choi et al.	5	276	1	189	20.0%^a^	68.5%	-	-
Couto et al.	50	141	34	53	68.0%	37.6%	-	4.50*,^b^ (1.90–10.4)
Donoghue et al.	136	164	102	116	75.0%	73.0%	-	-
Eisenberg et al.	199	240	144	170	72.4%	70.8%	1.08 (0.71–1.64)	1.23^c^ (0.78–1.94)
Ghedina et al.	62	168	57	124	91.9%	73.8%	-	-
Kaji et al.	331	294	279	219	84.0%	74.5%	1.80* (1.20–2.70)	1.80*,^d^ (1.00–3.10)
Miller et al.	946	295	708	174	74.8%	59.0%	2.05* (1.56–2.70)	2.01*,^e^ (1.48–2.73)
Pacheco et al.	275	218	202	142	73.5%	65.1%	-	-
Pallin et al.	51	994	48	823	94.1%	82.8%	-	3.40*,^f^ (1.50–7.60)

VL, videolaryngoscopy; DL, direct laryngoscopy; FPS, first-pass success; OR, odds ratio; *p < 0.05. Odds ratios given with 95% confidence intervals.

a Based only on 5 participants with one successful attempt.

b Adjusted for: second-year resident intubator, desaturation.

c Adjusted for: indication–coma/altered mental status, indication–critical airway obstruction, any difficult airway.

d Adjusted for: age < 2 years, body habitus, initial perceived airway difficulty, neck immobility, Mallampati score, mouth opening, thyromental distance, intubator level, rapid sequence intubation, trauma, and clustering.

e Adjusted for: age < 1 year, respiratory indication for intubation, laryngoscopist, and use of a neuromuscular blocking agent.

f Adjusted for: age < 1 year, sex, and use of a paralytic.

Adverse event data was reported for both VL and DL in six studies (Table 3) (18, 20, 22, 24–26). Adverse event rate ranged from 14.6 to 42.0% for VL and 13.9 to 41.3% for DL. Three studies reported unadjusted odds ratios for adverse events (22, 24, 25), with one study (33.3%) demonstrating significantly lower odds of adverse events with VL (25). Four studies calculated odds ratios adjusted for confounding factors and all of these studies found no significant difference between VL and DL for adverse events (18, 22, 24, 25). Various definitions of adverse events were used among the included studies, as shown in Table 4.

**Table 3:** Adverse event data.

Author	Total VL participants	Total DL participants	VL AE participants	DL AE participants	VL AE rate	DL AE rate	AE OR (unadjusted)	AE OR (adjusted)
Abid et al.	381	244	160	84	42.0%	34.4%	-	1.29^a^ (0.69–2.42)
Couto et al.	50	141	15	97	30.0%	68.8%	-	-
Eisenberg et al.	199	240	39	39	19.6%	16.3%	1.26 (0.77–2.05)	1.30^b^ (0.77–2.20)
Kaji et al.	328	294	48	41	14.6%	13.9%	1.10 (0.70–1.70)	0.80^c^ (0.40–1.40)
Miller et al.	946	295	269	112	28.4%	38.0%	0.65* (0.49–0.86)	0.74^d^ (0.51–1.08)
Pacheco et al.	275	218	98	90	35.6%	41.3%	-	-

VL, videolaryngoscopy; DL, direct laryngoscopy; AE, adverse event; OR, odds ratio; *p < 0.05. Odds ratios given with 95% confidence intervals.

a Adjusted for: sex, age, number of intubation attempts, difficult airway predictors, level of training of laryngoscopist, laryngoscope type, medication use, indications for intubation, and year.

b Adjusted for: indication–coma/altered mental status, indication–critical airway obstruction, any difficult airway.

c Adjusted for: age < 2 years, body habitus, initial perceived airway difficulty, neck immobility, Mallampati score, mouth opening, thyromental distance, intubator level, rapid sequence intubation, trauma, and clustering.

d Adjusted for: age < 1 year, respiratory indication for intubation, laryngoscopist, and use of a neuromuscular blocking agent.

**Table 4:** Adverse event definitions for studies included in meta-analysis.

Author	Defined adverse events
Abid et al.	Major: hypoxia (saturation < 90%), cardiac dysrhythmia or arrest, air leak (pneumothorax or pneumomediastinum), aspiration, oesophageal intubation with delayed recognition, and hypotension. Minor: dental trauma, mainstem intubation, oesophageal intubation with immediate recognition and mucosal injury.
Couto et al.	Desaturation (<80%) Severe: cardiac arrest, oesophageal intubation with delayed recognition, emesis with aspiration, hypotension requiring intervention, laryngospasm, pneumothorax, pneumomediastinum, and direct airway injury. Non-severe: mainstream bronchial intubation, oesophageal intubation with immediate recognition, emesis without aspiration, hypertension requiring therapy, epistaxis, dental or lip trauma, medication error, arrhythmia, and pain or agitation requiring additional medication or causing delay in intubation.
Eisenberg et al.	Cardiac arrest, dental trauma, direct airway injury (e.g., mucosal injury with bleeding), vomiting with or without aspiration, main-stem bronchus intubation, oesophageal intubation (immediate or delayed recognition), medication error, or hypotension.
Kaji et al.	Peri-intubation hypoxia (saturation < 90%), oesophageal intubation, vomiting, bradydysrhythmias, cardiac arrest, dental trauma, epistaxis, hypotension, lip laceration, laryngospasm, mainstem intubation, malignant hyperthermia, pneumothorax, tachydysrhythmias, tracheal tube cuff failure, medication errors, iatrogenic bleeding, and pharyngeal laceration.
Miller et al.	Aspiration, cardiac arrest, dysrhythmia, hypotension requiring intervention (fluid and/or vasopressors), hypoxia (moderate [saturation < 90%] or severe [saturation < 80%]), laryngospasm, lip or dental injury, mainstem bronchial intubation, mucosal injury, pneumothorax or pneumomediastinum, unrecognized oesophageal intubation with delayed recognition, and vomiting.
Pacheco et al.	Oesophageal intubation, mainstem intubation, aspiration, extubation, cuff damage, oxygen desaturation, pneumothorax, dental/airway trauma, hypotension, dysrhythmia, laryngospasm, medication error, and cardiac arrest.

### 3.4 Results of quality assessment

**Figure 2:**
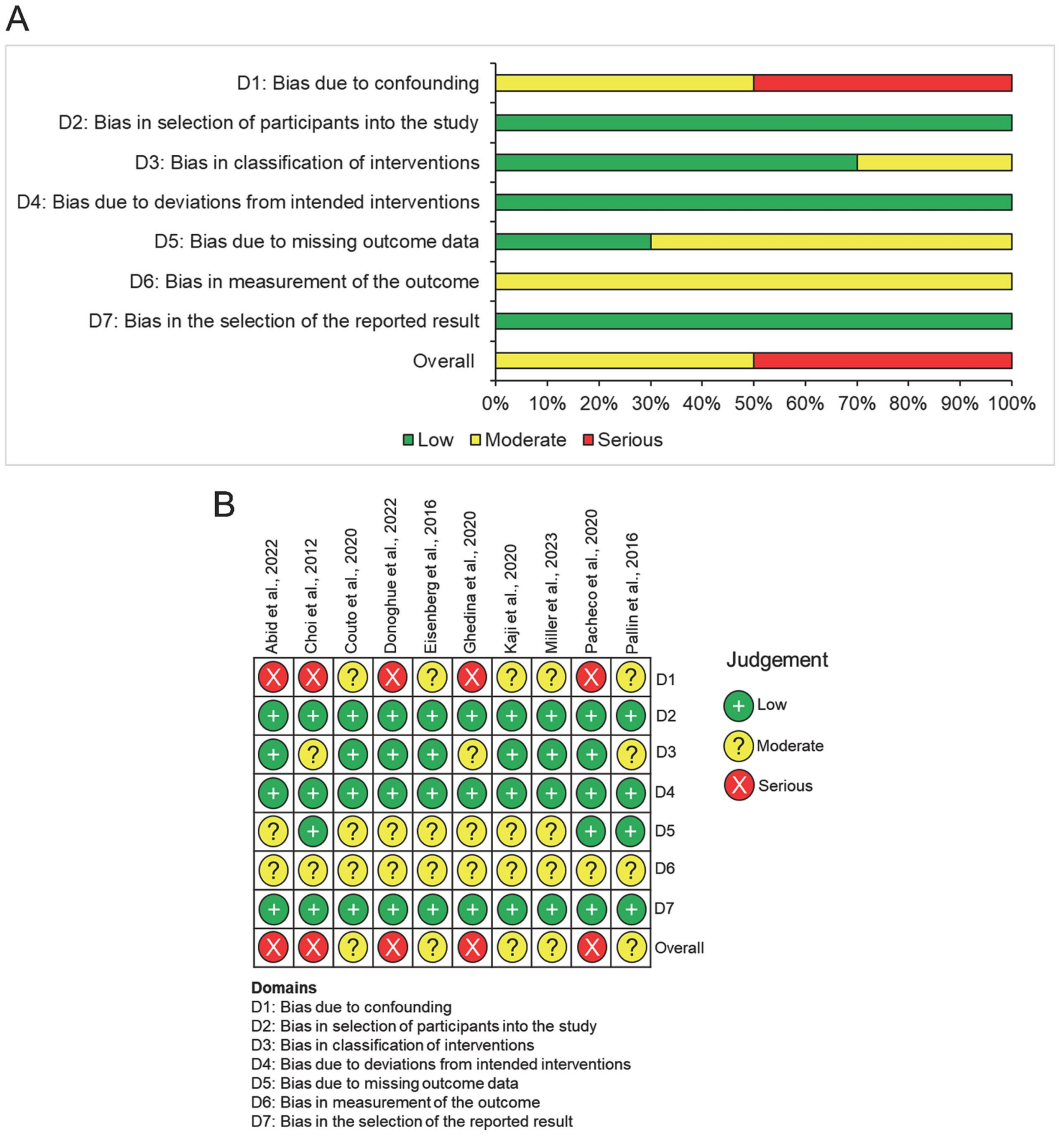
Quality assessment of included studies using risk of bias in non-randomised Studies of Interventions (ROBINS-I) tool. **(A)** Summary chart for the risk of bias in all included studies. **(B)** Risk of bias for each domain in individual studies.

All studies were assessed for risk of bias in the primary outcome using the ROBINS-I tool. Two reviewers undertook independent assessments and there was 100% interrater agreement for risk of bias judgements. Results for each bias domain and for the individual studies are presented in Figure 2. Overall risk of bias was judged to be serious for 5/10 (50%) studies (18, 19, 21, 23, 26), and moderate for the other five studies (20, 22, 24, 25, 27). Greatest risk was seen in bias due to confounding, with five studies being judged at serious risk of bias in this domain (18, 19, 21, 23, 26). Bias due to selection of participants, deviation from intended interventions, and selection of reported results was judged to be low risk in all studies. Bias due to measurement of the outcome was assessed as moderate risk in all studies given that it is not possible to blind operators or assessors to the method of laryngoscopy used.

### 3.5 Results of meta-analysis

**Figure 3:**
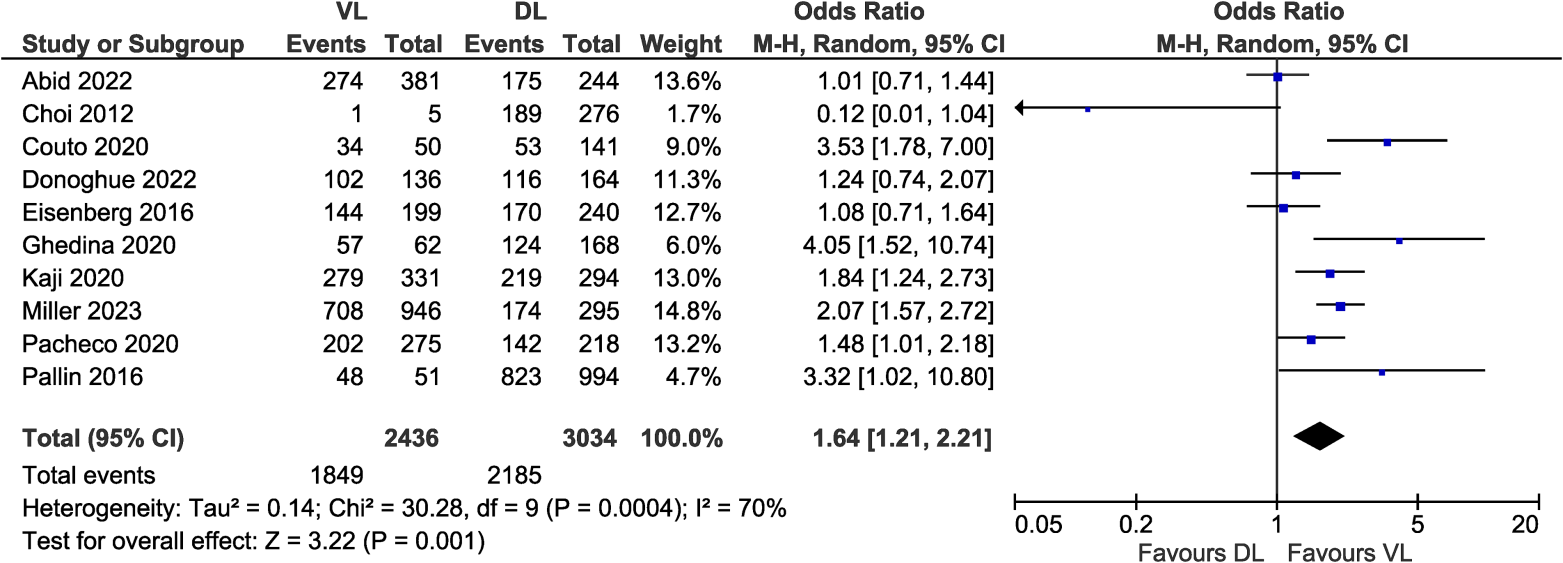
Meta-analysis of first-pass success data.

All studies reported FPS data for both VL and DL which were suitable for meta-analysis (Figure 3). Odds ratios for FPS with VL ranged from 0.12 to 4.05 across studies. The pooled odds ratio across all studies showed significantly increased odds of FPS with VL compared to DL (OR 1.64, 95% CI [1.21–2.21], *p* = 0.001) with moderate heterogeneity between studies (*I*^2^ = 70%).

**Figure 4:**
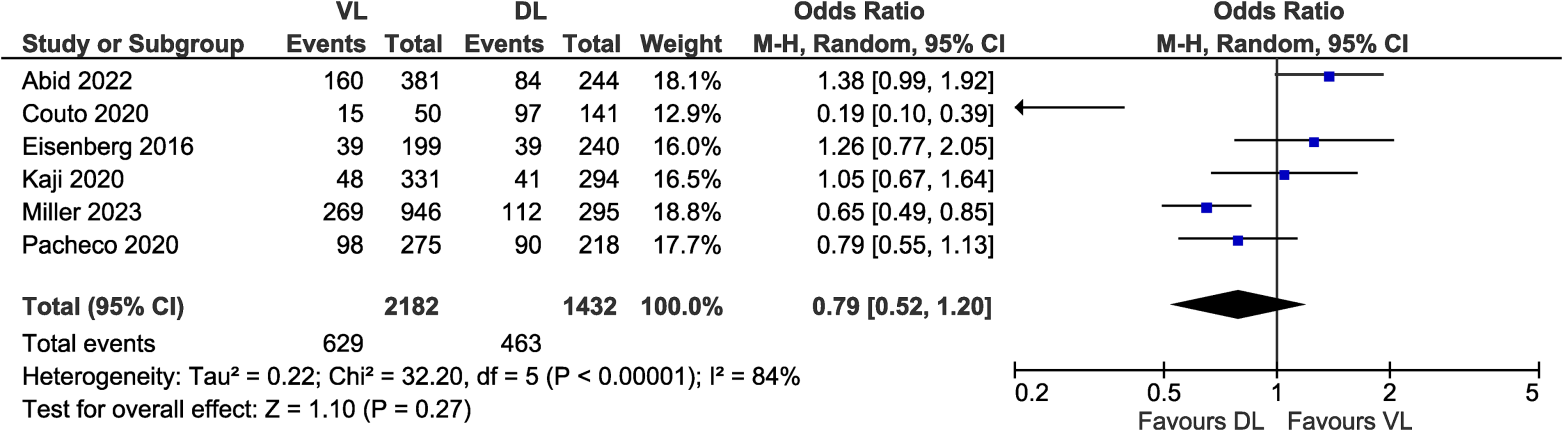
Meta-analysis of adverse event data.

Six studies were included in the meta-analysis for adverse events, representing 1,092 events across 3,614 intubations (Figure 4) (18, 20, 22, 24–26). Odds ratios for adverse events ranged from 0.19 to 1.38 across studies. The pooled odds ratio for these studies found no difference between VL and DL for odds of adverse events (OR 0.79, 95% CI [0.52–1.20], *p* = 0.27), with significant heterogeneity between studies (*I*^2^ = 84%).

## 4 Discussion

This systematic review demonstrated that there is limited evidence available on how the effectiveness and safety of VL compares to DL for intubation in the paediatric emergency setting. All studies found in the literature search were observational and were deemed to possess moderate to serious risk of bias, particularly due to the effect of confounding factors and in the measurement of outcomes. Meta-analysis of study outcomes indicated FPS is significantly more likely with VL than DL, however there was moderate heterogeneity between studies. Among studies reporting adverse effect data, there was significant heterogeneity and pooled outcomes suggested there is no difference in risk of adverse events between VL and DL.

### 4.1 First-pass success

Analysis of pooled FPS data from all studies has demonstrated VL to be beneficial over DL, however there was variability among the individual study results. Choi et al.’s study represented a significant outlier, with success on the first attempt in just 20.0% of VL cases (19), compared to 68.0–94.1% in the other studies. There are several factors which may have contributed to this low FPS rate. Firstly, there was a very low proportion of VL cases in their cohort (5/281), which led to an extremely small VL sample size which limits the validity of their result. Secondly, this study was published in 2012 and is the earliest study included in this review, at which time videolaryngoscopes were not widely available, therefore clinicians may have been unfamiliar with the technology and hence may represent a learning curve, or VL may have only been used for the most challenging cases. Couto et al. also reported the lowest FPS rate with DL among the studies, with only 37.6% of first attempts successful (20). This may have been influenced by a significantly greater proportion of their DL cohort being intubated by second-year residents compared to more experienced physicians (73% for DL compared to 54% for VL).

The results from this review suggest greater effectiveness of VL than has been previously demonstrated for intubation of adults in the emergency setting. Arulkumaran et al. found similar likelihood of FPS with VL and DL among 12 studies based in the emergency room (OR 1.25, 95% CI [0.96–1.62]), however FPS rates were significantly higher among novice and trainee clinicians using VL compared to DL (OR 1.95, 95% CI [1.45–2.64]) (28). Intubator training level varied across studies included in this review, with Couto et al. reporting 68.1% intubations being performed by second-year residents in a Brazilian emergency department (20), compared to a majority (63.0–81.0%) being undertaken by fellows in three US studies (18, 21, 22). This reflects national and international variation in emergency department staffing and airway management protocols.

Current RCT evidence of paediatric intubations in all settings has indicated significant reduction in failed first intubation attempts with VL in children up to 1 year old (OR 0.35, 95% CI [0.20–0.62]), but not in children of all ages (OR 0.78, 95% CI [0.41–1.47]) (13). For all studies in this review where age group data was reported, FPS rate was lowest in children <1 year of age (21, 23, 26, 27). Two studies demonstrated conflicting results regarding effectiveness of VL in infants, with Kaji et al. reporting double the odds of FPS with VL in children <2 years of age (OR 2.0, 95% CI [1.1–3.3]) (24), whereas Eisenberg et al. found significantly lower FPS in children <1 year of age when VL was used (OR 0.43, 95% CI [0.19–0.98]) (22).

### 4.2 Adverse events

There was significant heterogeneity between studies for this outcome, which is contributed to by the various definitions of adverse events across the studies (Table 4). Overall adverse event rate ranged from 14.3% in Kaji et al.’s study to 58.6% recorded by Couto et al. (20, 24), in keeping with the variability seen in other paediatric emergency studies (1, 2, 29). Pacheco et al. measured first-pass success without adverse events (FPS-AE) in their study, and suggested it should be the standard outcome measure for such studies given that uncomplicated intubation on the first attempt is the ultimate goal of emergency airway management (26). They recorded a FPS-AE rate of 60.0% with VL and 54.1% with DL, compared to FPS of 73.5 and 65.1%, respectively. Similarly, an Australian study by Long et al. reported a FPS rate of 78%, but only 49% without desaturation or hypotension (1). Abid et al. demonstrated how complication risk increases with number of intubation attempts, with adverse events more than three times more likely if two attempts are required compared to one (OR 3.26, 95% CI 2.11–5.03) (18). This highlights the importance of maximising FPS rate through interventions such as VL, to reduce the risk of adverse airway outcomes following ETI.

### 4.3 Further benefits of VL

Beyond offering improved FPS rates, VL can be a valuable teaching tool for emergency airway management. The video screen allows for visualisation of the airway anatomy by both the intubator and supervisor, giving the opportunity for real-time coaching which can significantly improve success rates (30). The ability to record video footage also allows for post-intubation debriefing and shared learning opportunities in later teaching sessions from a single patient encounter. Video recordings can further be used to extract data for research purposes as well as for quality assurance to ensure staff competency and patient safety (31). In pre-hospital and emergency medicine settings, VL could be used to facilitate tele-intubation and assist with airway management for critically unwell patients (8).

VL devices with Macintosh blades offer the benefit of giving operators the option for both direct and indirect visualisation of the glottis (6). As videolaryngoscopes become more widely available, they may become the recommended first-line device for ETI, with operators being able to begin intubating under direct vision, and switch to indirect visualisation on the video screen if difficulties arise, or if the glottic view is insufficient. Devices with hyperangulated blades which allow intubation without the degree of neck extension required for DL may then be more suitable for first-line use in individuals with a limited range of neck movement, such as in cases of trauma and certain congenital syndromes (4).

Although the Difficult Airway Society (DAS) guidelines from 2015 state that for unanticipated difficult tracheal intubation in children aged 1 to 8 years, more research is needed in the use of VL in paediatric clinical practice, our systematic review demonstrates higher FPS when using VL than DL and hence the evidence probably needs to be re-reviewed for paediatric use. In addition, VL is likely to be generalisable for other populations, different ethnicities, health conditions and even in instances where slight anatomical variations may exist. This is because VL technology provides an integrated camera to indirectly visualise the airway and avoid intubation where difficulties, for example, in visualising the glottis is anticipated. The American Society of Anaesthesiologists updated their guidelines for the management of the difficult airway in 2022 to include VL as a technique for intubation in paediatric patients but recommended that the choice of VL and other forms of intubation were to be based on previous experience, available resources, competency and context in which airway management will occur (32).

### 4.4 Limitations of included studies

As demonstrated in the quality assessment, risk of bias was high among all the studies included in this review. Given the observational nature of the research, there were often significant differences between baseline characteristics of the VL and DL groups, leading to bias due to confounders. Some studies included multivariate regression models to adjust for various factors such as age, training level of intubator, difficult airway characteristics, and ETI indication (20, 24, 25, 27, 29). Despite this, there may still be risk of confounding due to other undocumented factors that were not accounted for in these models. Randomisation of participants to either VL or DL in future studies would mitigate the risk of confounding bias, however, designing RCTs for emergency settings can be difficult due to issues identifying eligible participants, obtaining consent, and implementing a study protocol within the limited timeframe of emergency care (33). Despite these barriers, large-scale randomised trials comparing VL and DL in the emergency setting are underway, such as the US-based DEVICE trial (34).

Another major limitation is the lack of clarity in studies regarding how the videolaryngoscope is used. Given that devices with Macintosh blades (e.g., C-MAC) can be used for either direct or indirect laryngoscopy, it can be unclear to what extent the video technology is being used throughout the procedure. Seven of the included studies specifically defined VL as use of a videolaryngoscope regardless of how it was used by the operator (18, 20–22, 24–26). Donoghue et al. also used video review of intubations to determine whether there was video use (i.e., indirect laryngoscopy) during most of the glottic visualisation phase, most of the tube placement phase, or the entire procedure (21). Eisenberg et al. and Miller et al. chose to refer to use of a VL device as “video-assisted laryngoscopy” in order to encompass the variety of methods in which a videolaryngoscope can be used (22, 25).

Finally, the majority of studies in this review relied on data from written intubation documentation which has been shown to be significantly different from findings when video review is used (34). Donoghue et al. were the only authors to use videographic assessment as part of the Videography in Pediatric Resuscitation (VIPER) collaborative, which aims to provide a method of accurate examination of critical procedures such as ETI (21). Therefore, the data reported in the other studies may not be an accurate reflection of intubation outcomes. To improve the quality of the evidence for VL, we recommend that high quality, well controlled RCTs undertaken by suitably trained and equally competent persons to evaluate the performance of VL over DL in paediatric populations. This will help to eliminate the high risk of bias in future studies and avoid practitioner variabilities. In addition, there are a variety of devices used for VL and hence the same devices must be compared in these RCTs. We would also recommend the use of video to document the intubation procedure rather than just written notes to improve the accuracy of the notes and the intubation process.

### 4.5 Limitations of review processes

The conclusions drawn from this study are limited by the small body of evidence available, high heterogeneity between studies, and low quality of evidence. Selection bias may have occurred due to only including English language studies and not searching grey literature for unpublished studies, meaning that results are subject to publication and reporting bias. Interpretation of pooled effect measures from the meta-analyses should be considered in light of the moderate and high heterogeneity for FPS and adverse event data, respectively.

## 5 Conclusion

This review demonstrated that VL is associated with significantly greater FPS rate than DL for children intubated in emergency departments. This suggests that emergency departments should consider incorporating VL into airway management protocols to optimise intubation success on the first attempt. These results also provide a foundation to focus further randomised research and inform best clinical practice for intubation. VL devices can be valuable tools for teaching and quality assurance, as well as offering the opportunity for remote supervision of emergency ETI with real-time video sharing. However, current evidence for use of VL in this population is limited, and robust randomised trials are required which can adequately mitigate confounders of intubation success. Future studies should focus on the specific populations which may benefit most from VL, such as infants and those with difficult airways, and ensure clarity in defining how VL devices are used by operators.

## Data Availability

The original contributions presented in the study are included in the article/Supplementary material, further inquiries can be directed to the corresponding author.
