# Supplementary material for: Comparing the effectiveness and safety of videolaryngoscopy and direct laryngoscopy for endotracheal intubation in the paediatric emergency department: a systematic review and meta-analysis
Source: Front Med (Lausanne). 2024 Sep 19;11:1373460. doi: 10.3389/fmed.2024.1373460 (PMC11446787; doi:10.3389/fmed.2024.1373460)
Supplement: Supplementary file 1 [file Data_Sheet_1.DOCX]

Supplementary Material

# Database Searches

## MEDLINE

Database: Ovid MEDLINE(R) <1946 to May Week 1 2023>  
Search Strategy:  
1  exp Intubation, Intratracheal/ or exp Airway Management/ (131821)  
2  airway management.mp. (9223)  
3  intubat*.mp. [mp=title, book title, abstract, original title, name of substance word, subject heading word, floating sub-heading word, keyword heading word, organism supplementary concept word, protocol supplementary concept word, rare disease supplementary concept word, unique identifier, synonyms, population supplementary concept word, anatomy supplementary concept word] (87544)  
4  1 or 2 or 3 (173880)  
5  exp child/ or exp infant/ (2761824)  
6  exp Pediatrics/ (62929)  
7  ("child*" or "pediatric*" or "paediatric*" or "infant*" or "newborn*" or "neonate*").mp. [mp=title, book title, abstract, original title, name of substance word, subject heading word, floating sub-heading word, keyword heading word, organism supplementary concept word, protocol supplementary concept word, rare disease supplementary concept word, unique identifier, synonyms, population supplementary concept word, anatomy supplementary concept word] (3319434)  
8  5 or 6 or 7 (3320551)  
9  exp Laryngoscopes/ or exp Laryngoscopy/ (16030)  
10  ("videolaryngoscop*" or "video laryngoscope" or "video laryngoscopy").mp. [mp=title, book title, abstract, original title, name of substance word, subject heading word, floating sub-heading word, keyword heading word, organism supplementary concept word, protocol supplementary concept word, rare disease supplementary concept word, unique identifier, synonyms, population supplementary concept word, anatomy supplementary concept word] (2245)  
11  ("video-assisted laryngoscopy" or "video-assisted laryngoscope").mp. [mp=title, book title, abstract, original title, name of substance word, subject heading word, floating sub-heading word, keyword heading word, organism supplementary concept word, protocol supplementary concept word, rare disease supplementary concept word, unique identifier, synonyms, population supplementary concept word, anatomy supplementary concept word] (29)  
12  9 or 10 or 11 (16420)  
13  exp Emergency Service, Hospital/ (98248)  
14  exp Emergencies/ (43055)  
15  ("Emergency department" or "emergency" or "urgent").mp. [mp=title, book title, abstract, original title, name of substance word, subject heading word, floating sub-heading word, keyword heading word, organism supplementary concept word, protocol supplementary concept word, rare disease supplementary concept word, unique identifier, synonyms, population supplementary concept word, anatomy supplementary concept word] (404225)  
16  13 or 14 or 15 (430651)  
17  4 and 8 and 12 and 16 (207)  
18  limit 17 to english language (188)

## Embase

Database: Embase <1974 to 2023 May 10>  
Search Strategy:  
1  exp endotracheal intubation/ or exp intubation/ (120640)  
2  exp respiration control/ (20884)  
3  ("airway management" or "intubat*").mp. [mp=title, abstract, heading word, drug trade name, original title, device manufacturer, drug manufacturer, device trade name, keyword heading word, floating subheading word, candidate term word] (166692)  
4  1 or 2 or 3 (179198)  
5  exp child/ (3079137)  
6  exp pediatrics/ (127612)  
7  ("child*" or "pediatric*" or "paediatric*" or "infant*" or "newborn*" or "neonate*").mp. [mp=title, abstract, heading word, drug trade name, original title, device manufacturer, drug manufacturer, device trade name, keyword heading word, floating subheading word, candidate term word] (4068349)  
8  5 or 6 or 7 (4086517)  
9  exp videolaryngoscope/ (3683)  
10  ("videolaryngoscop*" or "video laryngoscope" or "video laryngoscopy").mp. [mp=title, abstract, heading word, drug trade name, original title, device manufacturer, drug manufacturer, device trade name, keyword heading word, floating subheading word, candidate term word] (6383)  
11  ("video-assisted laryngoscopy" or "video-assisted laryngoscope").mp. [mp=title, abstract, heading word, drug trade name, original title, device manufacturer, drug manufacturer, device trade name, keyword heading word, floating subheading word, candidate term word] (67)  
12  9 or 10 or 11 (6412)  
13  exp hospital emergency service/ or exp emergency health service/ or exp emergency/ or exp emergency treatment/ or exp emergency medicine/ or exp emergency care/ or exp pediatric emergency medicine/ or exp emergency ward/ or exp emergency patient/ (668991)  
14  ("Emergency department" or "emergency" or "urgent").mp. [mp=title, abstract, heading word, drug trade name, original title, device manufacturer, drug manufacturer, device trade name, keyword heading word, floating subheading word, candidate term word] (803401)  
15  13 or 14 (1002594)  
16  4 and 8 and 12 and 15 (310)  
17  limit 16 to english language (306)

## CENTRAL

#1 MeSH descriptor: [Intubation] explode all trees 6508

#2 MeSH descriptor: [Intubation, Intratracheal] explode all trees 5090

#3 MeSH descriptor: [Intubation, Intratracheal] explode all trees 5090

#4 “Intubation” OR “airway management” 22765

#5 #1 OR #2 OR #3 OR #4 23229

#6 MeSH descriptor: [Child] explode all trees 77718

#7 MeSH descriptor: [Pediatrics] explode all trees 1178

#8 “Child” OR “children” OR “paediatric” OR “pediatric” OR “infant” OR “newborn” OR “neonate” 235474

#9 #6 OR #7 OR #8 235531

#10 MeSH descriptor: [Laryngoscopes] explode all trees 672

#11 MeSH descriptor: [Laryngoscopy] explode all trees 1445

#12 “Videolaryngoscope” OR “videolaryngoscopy” OR “video laryngoscope” OR “video laryngoscopy” 1623

#13 #10 OR #11 OR #12 2900

#14 MeSH descriptor: [Emergencies] explode all trees 1667

#15 MeSH descriptor: [Emergency Medical Services] explode all trees 5216

#16 MeSH descriptor: [Emergency Service, Hospital] explode all trees 3314

#17 “Emergency department” OR “emergency” OR “urgent” 43936

#18 #14 OR #15 OR #16 OR #17 44625

#19 #5 AND #9 AND #13 AND #18 70

## Web of Science

# Web of Science Search Strategy (v0.1)

# Database: Web of Science Core Collection

# Searches:

1: (((ALL=(“Intubation” OR “airway management”)) AND ALL=(“Child” OR “children” OR “paediatric” OR “pediatric” OR “infant” OR “newborn” OR “neonate”)) AND ALL=(“Videolaryngoscopy” OR “videolaryngoscope” OR “video laryngoscopy” OR “video laryngoscope” OR “laryngoscopy” OR “laryngoscope”)) AND ALL=(“Emergency department” OR “emergency” OR “urgent”) Date Run: Thu May 11 2023 20:55:13 Results: 310
